# Fatty acid profiles recorded in ocean prey and California salmonine eggs reveal maternal ocean diets linked to thiamine deficiency

**DOI:** 10.1093/conphys/coag037

**Published:** 2026-06-10

**Authors:** Jarrod M Ludwig, Nathan J Mantua, Rachel C Johnson, John Field, Abigail E Ward, Freya E Rowland, Thomas H Williams, Taylor Lipscomb, Carson Jeffres, Jacques Rinchard

**Affiliations:** Department of Environmental Science and Ecology, State University of New York–Brockport, 350 New Campus Drive, Brockport, NY 14420, USA; Fish Ecology Division, Southwest Fisheries Science Center, NOAA Fisheries, 110 McAllister Way, Santa Cruz, CA 95060, USA; Fish Ecology Division, Southwest Fisheries Science Center, NOAA Fisheries, 110 McAllister Way, Santa Cruz, CA 95060, USA; Center for Watershed Science, University of California, Davis, 1 Shields Avenue, Davis, CA 95616, USA; Fish Ecology Division, Southwest Fisheries Science Center, NOAA Fisheries, 110 McAllister Way, Santa Cruz, CA 95060, USA; Center for Watershed Science, University of California, Davis, 1 Shields Avenue, Davis, CA 95616, USA; Columbia Environmental Research Center, U.S. Geological Survey, 4200 New Haven Road, Columbia, MO 65201, USA; Fish Ecology Division, Southwest Fisheries Science Center, NOAA Fisheries, 110 McAllister Way, Santa Cruz, CA 95060, USA; Department of the Interior, U.S. Fish and Wildlife Service–Headquarters, 5275 Leesburg Pike, Falls Church, VA 22041, USA; Center for Watershed Science, University of California, Davis, 1 Shields Avenue, Davis, CA 95616, USA; Department of Environmental Science and Ecology, State University of New York–Brockport, 350 New Campus Drive, Brockport, NY 14420, USA

**Keywords:** Diet, fatty acid, lipids, salmonine, trophic interactions, vitamin B_1_

## Abstract

Following the principle ‘you are what you eat’, fatty acid signatures (FASs) of adult fish tissues, when compared with those of potential prey, can link diet to nutritional status. In 2020, thiamine (vitamin B_1_) deficiency was diagnosed for the first time in multiple anadromous salmon populations in California. However, direct information linking ocean diet to thiamine status in marine-feeding salmonines is typically unavailable. This research aimed to link diet composition and egg total thiamine concentration of anadromous Chinook salmon, coho salmon and steelhead using FAS analysis. From 2020 to 2022, unfertilized eggs from these California salmonine species were collected alongside key forage species from the Pacific Ocean. FASs in eggs and prey were quantified using gas chromatography/mass spectrometry and total thiamine concentrations were quantified using high-performance liquid chromatography. Eggs rich in oleic acid (18:1n-9) reflected a diet dominated by Pacific herring and exhibited higher total thiamine concentrations. Conversely, eggs rich in eicosapentaenoic acid (20:5n-3) indicated a diet dominated by northern anchovy or euphausiid krill and were more likely to have low total thiamine concentrations. Polyunsaturated fatty acid proportions and the egg unsaturation index were strongly negatively correlated with egg total thiamine concentration, emphasizing the importance of lipid quality over quantity as a driver of egg thiamine concentration. This study highlights the utility of FASs for tracking the impacts of a changing ocean prey base on diet and underscores how dietary shifts can directly impact egg thiamine status in anadromous salmonines.

## Introduction

Relative concentrations of fatty acids, or fatty acid signatures (FASs), are transferred from prey to predator, serving as dietary tracers following the principle ‘you are what you eat’ (Lovern, 1935). FASs have been widely used to elucidate predator–prey relationships, including in salmonine fishes (Lovern, 1935; Napolitano, 1999; Dalsgaard *et al*., 2003; Daly *et al*., 2010; Happel *et al*., 2017). Because a predator’s diet determines the fatty acids available in the body, egg fatty acid composition may reflect those accumulated by the female during vitellogenesis. Laboratory studies have shown that maternal diet can directly influence both lipid content and FASs of fish eggs (Rainuzzo, 1993; Fernandez-Palacios *et al*., 1995; Navas *et al*., 1997; Rodriguez *et al*., 1998; Pickova *et al*., 1999). Beyond diet reconstruction, FASs provide insight into egg quality (Pickova *et al*., 1998; Sargent *et al*., 1999; Izquierdo *et al*., 2001). Many polyunsaturated fatty acids (PUFAs) are required during early development, particularly for nervous system formation (Pickova *et al*., 1999). The unsaturation index (UI), which reflects the relative proportion of fatty acids with varying degrees of unsaturation, is commonly used as an indicator of lipid quality (Chen and Liu, 2020). Higher UI values likely indicate greater proportions of PUFAs, which can have beneficial and adverse effects on fish health.

In 2020, thiamine deficiency complex (TDC) was documented in California’s anadromous Chinook salmon (Mantua *et al*., 2021, 2025). TDC is a nutritional deficiency of thiamine (vitamin B_1_) that causes a range of health effects, including neurological problems and death. Because animals cannot synthesize thiamine, they must acquire it from their diet and therefore are especially vulnerable to TDC. When adult females produce eggs with insufficient thiamine reserves, their offspring may experience early mortality during or after the yolk-sac fry phase, also referred to as a free embryo (Halver, 1972; Balon, 1975; Morito *et al*., 1986). Early mortality due to TDC has been documented in salmonine species such as lake trout (*Salvelinus namaycush*) in the Laurentian Great Lakes and Atlantic salmon (*Salmo salar*) in the Baltic Sea, where it has been linked to recruitment failure (Brown *et al*., 1998; Honeyfield *et al*., 2005; Keinänen *et al*., 2012, 2025). With many of California’s salmonine populations listed as threatened or endangered on a state and federal level, further hindrances to population recovery such as TDC need to be assessed and resolved for conservation success.

Two main hypotheses have been proposed to explain TDC in salmonine species: (i) the consumption of prey containing thiaminase, an enzyme that degrades thiamine, and (ii) diets rich in lipids and unsaturated fatty acids (Honeyfield *et al*., 2005; Keinänen *et al*., 2022; Richter *et al*., 2023). Predators consuming prey rich in thiaminase can lose thiamine via thiaminolytic activity in the prey before it digests completely, leading to thiamine deficiency (Green, 1936; Wolf, 1942; Honeyfield *et al*., 2005). The second hypothesis suggests an inverse relationship between prey fat content and thiamine concentrations (Keinänen *et al*., 2012, 2018, 2025). As thiamine is essential for energy metabolism, predators consuming lipid-rich prey require more thiamine. Furthermore, unsaturated fatty acids are particularly susceptible to lipid peroxidation (Lehninger, 1970; Alvarez *et al*., 1998). Alewife (*Alosa pseudoharengus*), a prey species high in both thiaminase activity and lipids, has been associated with TDC in Great Lakes lake trout (Greig and Gnaedinger, 1971; Fitzsimons and Brown, 1998; Honeyfield *et al*., 2005). Northern anchovy in the Pacific Ocean (*Engraulis mordax*) is high in both thiaminase activity and lipids and has a low thiamine content. Stomach content analysis linked anchovy-dominated diets with TDC in California Chinook salmon (Mantua *et al*., 2025). Therefore, diet quality has important implications for predator health.

California Chinook salmon typically consume a diverse diet composed primarily of euphausiid krill, pelagic juvenile rockfish (*Sebastes* spp*.*), market squid (*Doryteuthis opalescens*), Pacific herring (*Clupea pallasii*), sardines (*Sardinops sagax*) and northern anchovy (*E. mordax*) (Merkel, 1957; Thayer *et al*., 2014). These taxa are also key forage for numerous predatory fishes, seabirds and marine mammals throughout the California Current ecosystem (Szoboszlai *et al*., 2015). Most of these prey taxa have strong seasonal, interannual and interdecadal variability in abundance in central California marine waters as reflected in survey data and historical Chinook salmon diets (MacCall, 1996; Schwartzlose *et al*., 1999; Ralston *et al*., 2013; Thompson *et al*., 2017; Santora *et al*., 2021; Suca *et al*., 2022). Coho salmon (*Oncorhynchus kisutch*) diet data are very similar to those of Chinook salmon, but with greater reliance on marine pelagic invertebrates like amphipods, market squid and crab megalopae (*Cancer* spp*.*) (Groot and Margolis, 1991). Steelhead (anadromous *Oncorhynchus mykiss*), known for their diverse ocean migrations and surface-feeding behaviour, consume primarily gonatid squid, euphausiids and forage fish such as northern anchovy (Teo *et al*., 2013; Hayes *et al*., 2016; Myers, 2018).

A regime shift in the Central California Current Ecosystem’s prey community was seen in recent years through a dramatic increase (~400%) in northern anchovy abundance between 2015 and 2020, levels not observed since the early 1970s (Hinchliffe *et al*., 2025). Assessing maternal diet is essential to understanding the sources of variation in egg total thiamine concentrations. However, there has been no direct link established between Chinook salmon ocean diet and the thiamine status of their bodies or their eggs. Moreover, ocean diet data for California coho salmon (fishery closed since 1993) and steelhead (not targeted by ocean fisheries) are unavailable.

To address the knowledge gap of salmonine ocean diet and egg thiamine status, we used FAS analysis of both potential prey items and anadromous Pacific salmon and steelhead eggs at spawning time. Specifically, our objectives were to (i) determine the diet of California Chinook salmon, coho salmon and steelhead using egg FASs to address the hypothesis of a diet dominated by the abundant northern anchovy, and (ii) link the diet of individual fish to their corresponding egg total thiamine concentration to address the hypothesis of poor nutritional quality caused by a diet dominated by northern anchovy, and (iii) compare the diet-thiamine linkages among individuals, populations and species across three consecutive years of data collection to address the hypothesis of feeding patterns across space and time.

## Materials and methods

### Sample collection and processing

**Figure 1:**
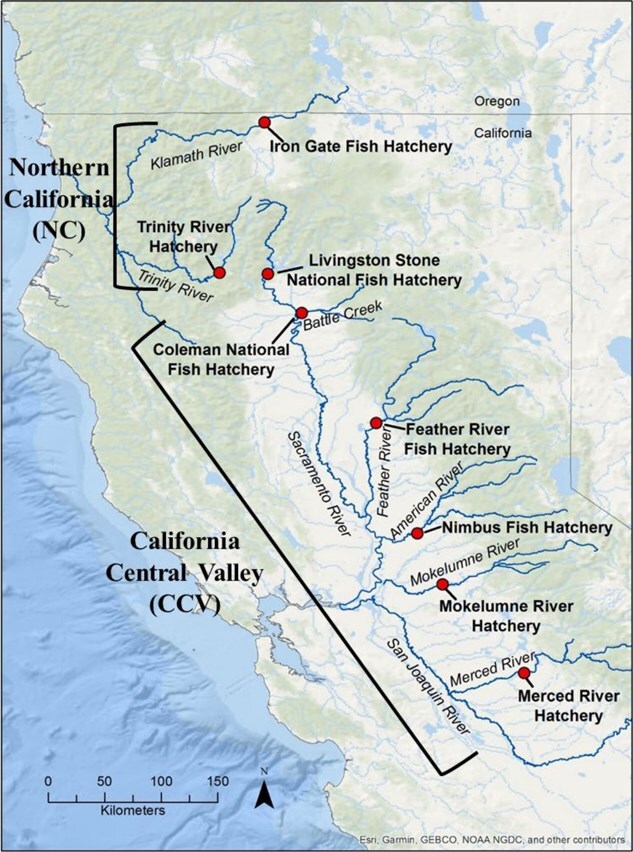
Map of hatcheries in California where Chinook salmon (*O. tshawytscha*), coho salmon (*O. kisutch*) and steelhead (*O. mykiss*) eggs were collected during their respective spawning runs from 2020 to 2022. California Central Valley (CCV) hatcheries: livingston stone, coleman, feather, nimbus, mokelumne and merced. Northern California (NC) hatcheries: iron gate and Trinity.

**Figure 2:**
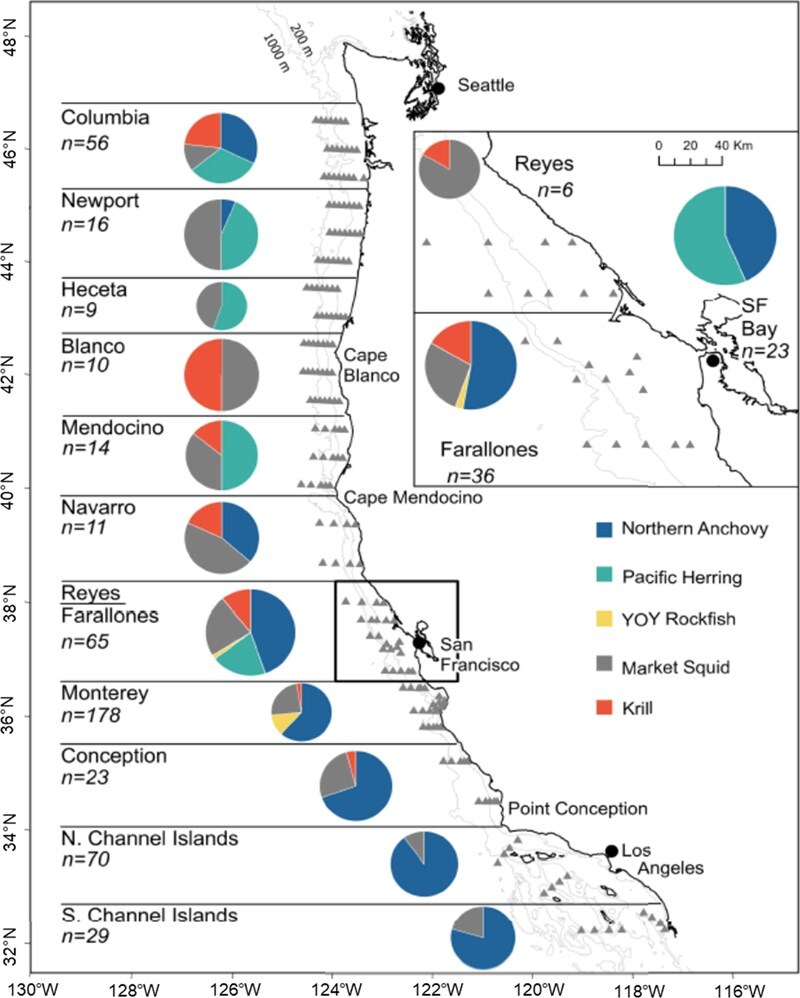
Map of midwater trawl locations in 2020–2022 for potential prey forage species of Chinook salmon, coho salmon and steelhead. Midwater trawl locations are denoted by grey triangles. The proportion of each species caught in the survey region is indicated by the pie chart and the total number of individuals (*n*) caught from all species is denoted by region.

Unfertilized Chinook salmon and coho salmon eggs were collected during 2020–2022 and unfertilized steelhead eggs during 2021–2022 from six hatcheries in California’s Central Valley (CCV) on the Sacramento/San Joaquin River system, four of which are multi-species hatcheries. Eggs were also collected from two multi-species hatcheries in northern California (NC) on the Klamath River system (Fig. 1). In 2020–2022, potential prey species were collected as part of the Rockfish Recruitment and Ecosystem Assessment Survey, which completed forage surveys along the West Coast ranging from the South Channel Islands, CA, to central WA using midwater trawls from May through June (Field *et al*., 2021; Fig. 2). A variety of prey species were collected and immediately frozen at −80°C during the surveys; however, only northern anchovy, market squid, euphausiid krill (a mix of *Thysanoessa spinifera* and *Euphausia pacifica*, see Daly *et al*., 2010 for FAS comparison), juvenile/young-of-year (YOY) rockfish and Pacific herring were analysed in this study. These species have been the recent prominent prey of Chinook salmon populations found in central California waters, as described above (see also Thayer *et al*., 2014; Wells *et al*., 2024). Each individual prey (besides krill, as multiple individuals were grouped) was measured and weighed before being homogenized individually (whole body) in a lab-grade stainless steel blender with dry ice. The homogenized sample was then divided for thiamine, lipid and fatty acid, and thiaminase analyses.

### Animal ethics

All eggs were collected under a permit approved by the California Department of Fish and Wildlife (Request 25013) and the NOAA (NMFS Sec. 10(a)(1)(A): 17299-3M).

### Lipid and fatty acid analyses

Lipids were extracted from egg (~1 g) and prey homogenate samples (~1 g) using a 2:1 chloroform-to-methanol solvent containing 0.01% butylated hydroxytoluene to prevent lipid oxidation (Folch *et al*., 1957). Lipid content (%) was calculated as grams of lipids extracted per gram of sample. Individual egg mass was not measured because eggs were processed as pooled, homogenized samples to obtain sufficient material for lipid extraction and fatty acid analysis; thus, lipid content is reported on a mass-normalized (wet weight) basis rather than per egg. This approach is appropriate for assessing overall biochemical composition and facilitates comparisons among samples independent of variation in individual egg size. Nonadecanoate acid (19:0) was added as an internal standard based on the amount of total lipids present (8/50 mg of lipids). Lipids were then transmethylated to create fatty acid methyl esters (FAMEs; Metcalfe and Schmitz, 1961). FAMEs were analysed using an Agilent Technologies 7890A gas chromatograph system with an Agilent Technologies 7693 autosampler and an Agilent Technologies 5975C inert XL EI/CI mass selective detector with a triple-axis detector (Agilent Technologies Inc., Santa Clara, CA). An Agilent J & WGC column with 30 m × 0.250 mm × 0.50 μm thickness was used with helium as the carrier gas. Run conditions were set following Happel *et al*. (2017). Individual FAMEs were identified using fragment ions and by comparing their retention times to those of authentic standard mixtures (FAME mix 37 components, Sigma-Aldrich, St. Louis, MO) with known spectrographic patterns of FAMEs. The proportions of individual FAMEs relative to the total amount of FAMEs detected were determined. The UI was calculated from the individual egg FAME proportions following the formula by Chen and Liu (2020): UI = 1 × (sum of fatty acids with 1 double bond (%); monoenoics) + 2 × (sum of fatty acids with 2 double bonds (%); dienoics) + 3 × (sum of fatty acids with 3 double bonds (%); trienoics) + 4 × (sum of fatty acids with 4 double bonds (%); tetraenoics) + 5 × (sum of fatty acids with 5 double bonds (%); pentaenoics) + 6 × (sum of fatty acids with 6 double bonds (%); hexaenoics)

### Thiamine analysis

Thiamine was extracted from egg (~1 g) and prey homogenate (~1 g) samples following the methods of Brown *et al*. (1998). After thiamine extraction, thiamine was quantified using high-performance liquid chromatography (Agilent Technologies 1200 series, Agilent Technologies Inc.) according to Brown *et al*. (1998), modified by Futia *et al*. (2017). Three vitamers of thiamine (free thiamine—TH, thiamine monophosphate—TMP, thiamine pyrophosphate—TPP) were identified and their concentrations were quantified using a standard curve for each. The concentrations of TH, TMP and TPP were summed to express the total thiamine concentration in nmol/g of egg or prey, which avoids bias associated with variation in individual egg or prey size.

Total thiamine concentrations that induce mortality (effect concentration for 50% of the population or EC50) in California Chinook salmon eggs were evaluated using a dose–response model from laboratory rearing data to estimate total thiamine-dependent free embryo and alevin mortality (Bell, 2022; Mantua *et al*., 2025; Table 1). While total thiamine-dependent survivorship experiments have not been conducted for California coho salmon or steelhead, laboratory rearing data investigating free embryo mortality caused by TDC have been collected for these species in Lake Ontario (Futia and Rinchard, 2019). As Great Lakes Chinook salmon and coho salmon total thiamine-induced mortality rate thresholds are similar, the same thresholds for California Chinook salmon are used for California coho salmon. Using steelhead egg total thiamine and associated free embryo mortality data collected from Futia and Rinchard (2019), a dose–response model was used to establish California steelhead egg total thiamine mortality rate thresholds (Table 1).

**Table 1:** Egg total thiamine thresholds used to evaluate the occurrence of TDC in the offspring of Chinook salmon (*Oncorhynchus tshawytscha*), coho salmon (*O. kisutch*) and steelhead (*O. mykiss*) resulting in offspring mortality

**Thiamine assessment (nmol/g)**				
**Species**	**Severely impacted**	**Impacted**	**Likely impacted**	**Unlikely impacted**
**Chinook salmon**	<2.7	2.7–5.9	5.9–7.7	>7.7
**Coho salmon**	<2.7	2.7–5.9	5.9–7.7	>7.7
**Steelhead trout**	<5.3	5.3–7.1	7.1–7.9	>7.9

Severely impacted represents the EC_50_ (50% of offspring affected by TDC), impacted represents the EC_10_ (10% of offspring affected by TDC), likely impacted represents the EC_5_ (5% of offspring affected by TDC) and unlikely impacted represents <5% of offspring affected by TDC.

### Thiaminase activity assays

Corresponding thiaminase activity data from individual prey were obtained from Mantua *et al*. (2025). The method for the thiaminase activity assay has been described in Rowland *et al*. (2026) but is briefly described here. The methods of Hanes *et al*. (2007) and Kraft *et al*. (2014) were followed to measure thiaminase I activity using a 4-nitrothiophenol assay on prey homogenate samples. Prey homogenate (0.5 g) was suspended in phosphate buffer (1.25 ml of 100 mM, pH 6.5) then bead-beaten to lyse cells and centrifuged at 4°C for 10 min (16 000× g for 10 min). Supernatants were assayed using Tris (2-carboxyethyl) phosphine hydrochloride (TCEP, 10 mM, pH 6.9) buffer with 4-nitrothiophenol (80 μM), both without (400 μM) and with thiamine, in microtiter plates. The microtiter plate wells were filled with 97 μL of solution using a multi-channel pipette, including three wells with solution with additional thiamine and three without thiamine. Enzymatic reactions were initiated by adding 3 μL of sample supernatant to each well. Absorbance readings at 411 nm were taken every minute for 60 min at 37°C in a Synergy HT Multi-Detection Microplate Reader (BioTek Instruments, Winooski, VT). Thiaminase activity was calculated by linear regression (Microsoft Excel software) of the change in absorbance over time and corrected for non-thiaminase activity by the difference in slopes (with and without thiamine added). Data were then converted to concentration using the molar extinction coefficient for 4-nitrothiophenol (13 650 M^−1^ cm^−1^) and reported as nmol/min/g-sample. All assays were performed in triplicate. Solution blanks and positive control samples (*Paenibacillus thiaminolyticus*) were run with each plate of samples as a QA/QC check.

### Statistical analysis

Multivariate statistical analyses were conducted in Primer (Plymouth Routines in Multivariate Ecological Research, V7) and univariate analyses using Program R. To assess differences in prey FAS among species, a permutational multivariate analysis of variance (PERMANOVA) was used. To identify influential fatty acids causing variation among prey species, a principal component analysis (PCA) was performed. Lastly, a similarity percentage analysis (SIMPER) was used to determine the contribution of each fatty acid to the dissimilarity among species as well as the overall percent dissimilarity among prey species. Prey total thiamine concentration, lipid content and thiaminase activity were evaluated for normality using a Shapiro–Wilk test on model residuals of the raw data. These data were determined to be not normal and therefore a Kruskal–Wallis (KW) with Dunn’s post-hoc test was used to evaluate differences among prey species.

A similar analysis was conducted to determine differences among egg FAS based on species, run and location (PERMANOVA and PCA). Differences in individual egg FAS indicated variation in primary prey species consumption. A canonical analysis of principal coordinates was used to show misclassifications of egg samples among hatcheries based on the FAS. This analysis was performed on all species and Chinook salmon runs, besides late fall-run and winter-run, as at least two groups (hatcheries) are needed for group classification.

A correlation analysis was used to link egg FAs, lipid content and UI with egg total thiamine concentrations. A Shapiro–Wilk test on the model residuals of the raw data determined that these data were not normal. Therefore, a Spearman’s rank test was used to evaluate the strength and direction of the correlation. To identify significant differences in egg lipid content, a KW or Mann–Whitney *U* (MW) test was used. All means are provided with their SDs.

### Ethical declarations

All samples were collected at California or federal (USFWS) hatcheries and permitted by the California Department of Fish and Game and NOAA Fisheries for analysis.

## Results

### Thiamine concentrations, lipid contents and fatty acid proportions of prey

Yearly differences in prey nutritional analyses were not considered, as differences were greater among species. Total thiamine concentrations were significantly different among prey species (KW: *H* = 197.97, *df* = 4, *P* < 0.001; Table 2). Market squid had the highest total thiamine concentration, meanwhile northern anchovy and Pacific herring had the lowest. Krill and rockfish total thiamine concentrations did not significantly differ from each other (KW: *P* = 0.227). Thiaminase activity was significantly different among prey species (KW: *H* = 210.70, *df* = 4, *P* < 0.001; Table 2). The greatest thiaminase activity was found in anchovy, followed by Pacific herring. However, thiaminase activity did not differ significantly among market squid, rockfish and krill (KW: *P* > 0.05). Lipid content was significantly different among prey species (KW: *H* = 264.80, *df* = 4, *P* < 0.001; Table 2). Northern anchovy and Pacific herring had the highest lipid content, and krill had the lowest. Rockfish and market squid lipid content did not significantly differ (KW: *P* = 0.086). Pooled data for all species revealed that total thiamine concentration was negatively correlated with lipid content and thiaminase activity (Spearman’s rank: lipid content: *r_s_* = −0.445, *P* < 0.001; thiaminase activity: *r_s_* = −0.412, *P* < 0.001).

**Table 2:** Mean ± standard deviation (range) of total thiamine concentration (nmol/g), thiaminase activity (nmol/min/g) and whole-body lipid content (%) of the main prey species collected from the Pacific Ocean in 2020–2022

**Species**	**Northern anchovy**	**Pacific herring**	**Krill**	**Market squid**	**Rockfish**
**Sample size (*N*)**	265	50	35	109	22
**Thiamine concentration (nmol/g)**	1.94 ± 1.50^c^ (0.3–9.7)	2.97 ± 3.06^b^ (0.4–16.9)	4.64 ± 3.50^d^ (0.5–13.5)	7.75 ± 4.55^a^ (0.7–21.0)	4.97 ± 2.31^d^ (1.5–8.8)
**Thiaminase activity (nmol/min/g)**	28.78 ± 29.89^a^ (2.3–206.0)	6.57 ± 7.93^b^ (0.3–37.0)	1.11 ± 1.66^c^ (0.0–4.3)	2.97 ± 3.23^c^ (0.0–16.7)	2.57 ± 3.88^c^ (0.0–11.0)
**Lipid content (%)**	7.22 ± 3.99^a^ (1.7–19.2)	5.96 ± 3.03^b^ (1.3–13.2)	1.59 ± 0.60^d^ (0.9–4.6)	2.52 ± 0.42^c^ (1.9–4.9)	2.45 ± 0.79^c^ (1.5–4.9)

Sample sizes (*N*) analysed for each species are provided. Superscript letters represent significant differences (p < 0.05) between species.

**Figure 3:**
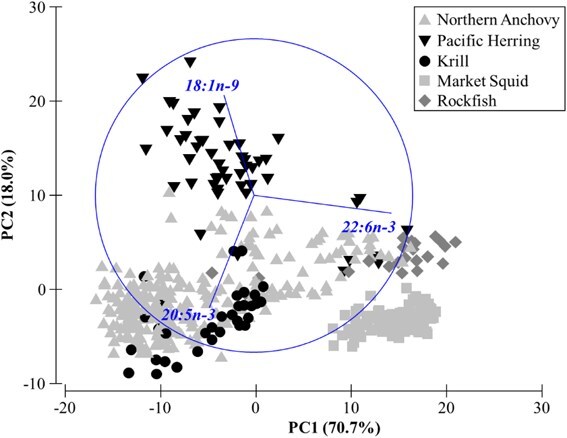
PCA of main prey species FASs collected from 2020 to 2022 based on the proportions of fatty acids. Vectors for 18:1n-9 (oleic acid), 20:5n-3 (EPA) and 22:6n-3 (DHA) are included based on their loading values. The percent variation accounted for by each principal component (PC) is included in parentheses on the axis title.

**Figure 4:**
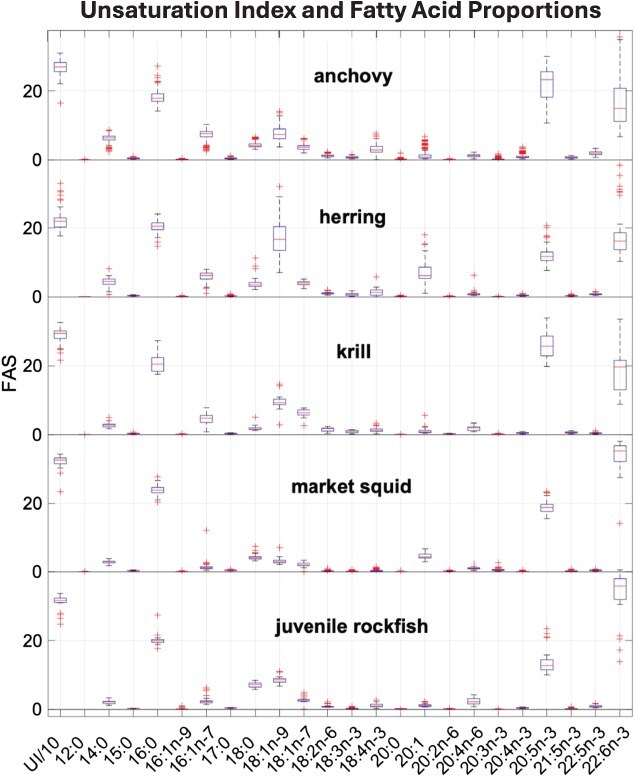
UI (divided by 10) and fatty acid proportions (%) for main prey species collected from 2020 to 2022. On each box, the central mark indicates the median, and the bottom and top edges of the box indicate the 25th and 75th percentiles, respectively. The whiskers extend to the most extreme data points not considered outliers, and the outliers are plotted individually using the ‘+’ marker symbol.

There was a significant difference in FAS among prey species (PERMANOVA: Pseudo-F = 241.84, *df* = 4, *P* < 0.001; Tables S1 and S2). PCA determined that rockfish and market squid were characterized by a high proportion of docosahexaenoic acid (DHA; *X̅* = 34.1 and 34.5%, respectively), northern anchovy and krill by high proportions of eicosapentaenoic acid (EPA; *X̅* = 22.0 and 26.3%, respectively), and Pacific herring by an elevated oleic acid proportion (18:1n-9; *X̅* = 17.1%) (Fig. 3). Therefore, the fatty acids DHA, EPA and oleic acid were retained in the egg fatty acid analysis as they characterized each prey species and were responsible for the significant differences in their FASs (Fig. 4).

### Thiamine concentrations, lipid contents and fatty acid proportions of *Chinook Salmon* eggs

**Figure 5:**
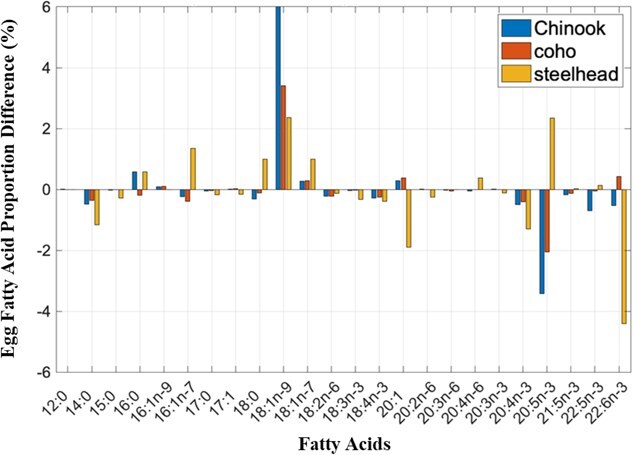
Differences in median egg FAS proportions between eggs classified as high (>8 nmol/g) and low (<8 nmol/g) total thiamine for Chinook salmon (*O. tshawytscha*), coho salmon (*O. kisutch*) and steelhead (*O. mykiss*).

The proportions of the fatty acids DHA, EPA and oleic acid in Chinook salmon eggs were responsible for significant differences among egg FASs. Eggs with low total thiamine (<8 nmol/g) were primarily rich in EPA (Fig. 5). Eggs with high in total thiamine (<8 nmol/g) were primarily rich in oleic acid (Fig. 5). Therefore, since differences in fatty acid proportions were present, the diet of Chinook salmon likely explains the presence or absence of thiamine deficiency.

#### Fall-run

Eggs from fall-run Chinook salmon were collected at five CCV hatcheries (Coleman, Feather, Nimbus, Mokelumne and Merced) and two NC hatcheries (Trinity and Iron Gate) (Table 3). Of all fall-run Chinook salmon eggs sampled in CCV, 22% were impacted, 32% were likely impacted, and 46% were unlikely impacted by TDC (Table 4). In NC, fall-run Chinook salmon egg total thiamine concentrations were higher than those from CCV, with 88% of eggs unlikely to be impacted by TDC. Also, CCV eggs had a greater lipid content than NC eggs (11.42 ± 1.02% vs. 10.16 ± 1.17%; MW: *U* = 62 722, *P* < 0.001).

**Table 3:** Mean ± standard deviation of Chinook salmon (*O. tshawytscha*) egg total thiamine concentrations (nmol/g) for unfertilized eggs collected during 2020–2022. Sample size (*N*) is provided for each run per hatchery and year

		**California’s Central Valley (CCV)**												**Northern California (NC)**			
**Species**	**Year**	***N***	**Coleman**	***N***	**Feather**	***N***	**Nimbus**	***N***	**Mokelumne**	***N***	**Merced**	***N***	**Livingston**	***N***	**Trinity**	***N***	**Iron gate**
Chinook fall-run	2020	30	11.71 ± 4.37	30	11.41 ± 4.89	30	10.04 ± 3.65	31	11.44 ± 4.44	30	13.99 ± 5.37	0		30	16.36 ± 4.09	30	19.03 ± 4.41
	2021	30	4.85 ± 1.60	30	7.04 ± 3.17	30	6.43 ± 2.10	30	8.23 ± 3.49	28	6.35 ± 2.97	0		30	12.20 ± 3.32	30	12.17 ± 3.71
	2022	30	8.17 ± 4.24	30	7.77 ± 3.96	30	6.28 ± 3.65	30	9.14 ± 5.47	12	9.63 ± 6.04	0		30	10.07 ± 3.59	30	12.35 ± 3.53
Chinook late fall-run	2020	33	4.91 ± 2.08	0		0		0		0		0		0		0	
	2021	30	7.64 ± 2.28	0		0		0		0		0		0		0	
	2022	41	5.06 ± 1.45	0		0		0		0		0		0		0	
Chinook winter-run	2020	0		0		0		0		0		29	5.18 ± 4.39	0		0	
	2021	0		0		0		0		0		29	3.13 ± 1.53	0		0	
	2022	0		0		0		0		0		28	3.41 ± 3.69	0		0	
Chinook spring-run	2020	0		30	9.21 ± 4.84	0		0		0		0		30	13.08 ± 2.46	0	
	2021	0		38	9.86 ± 5.04	0		0		0		0		31	14.84 ± 2.71	0	
	2022	0		30	8.75 ± 5.60	0		0		0		0		30	13.79 ± 3.24	0	

A sample size of ‘0’ for all years indicates that species/run is not received at the given hatchery.

**Table 4:** Percentage of sampled unfertilized eggs in each of the established total thiamine status classifications for each salmonine population

**Species**	**Sample size (*N*)**	**Severely impacted**	**Impacted**	**Likely impacted**	**Unlikely impacted**
CCV Chinook fall-run	431	3	19	32	46
NC Chinook fall-run	180	0	1	11	88
Chinook late fall-run	104	6	34	47	13
Chinook winter-run	86	49	35	11	5
CCV Chinook spring-run	98	6	17	24	53
NC Chinook spring-run	91	0	0	2	98
Coho	186	8	23	17	52
CCV steelhead	125	24	13	12	51
NC steelhead	60	5	8	5	82

Populations denoted with ‘CCV’ are from California’s Central Valley hatcheries and with ‘NC’ are from Northern California hatcheries. All Chinook late fall-run and winter-run are from CCV and all coho are from NC. Sample size is the number of females with eggs collected for thiamine analysis.

Fall-run Chinook salmon egg FASs were significantly different between regions (PERMANOVA: Pseudo-F = 154.53, *df* = 1, *P* < 0.001; Tables S3 and S4). PCA results identified that most CCV eggs were rich in EPA, whereas most eggs from NC were rich in oleic acid (Fig. S1A). A canonical analysis of principal coordinates on egg FAS (*N* = 611) determined that 39% of eggs were correctly assigned to the hatchery they were collected from. Of those misclassified, 68% were eggs collected from a CCV hatchery but their egg FAS resembled those of another CCV hatchery (*N* = 253 out of 373). Ten percent of eggs were from CCV populations that had an FAS resembling those of NC populations (rich in oleic acid); these eggs also presented higher total thiamine concentrations (Fig. S1B).

Comparing prey and egg FAS, most CCV fall-run Chinook salmon eggs had high proportions of EPA, indicating a diet of northern anchovy or krill. Most eggs collected from NC hatcheries were rich in oleic acid, indicating the consumption of Pacific herring. However, not all CCV fish had the same egg FASs, which suggests differences in diet, with a few having greater similarities to NC egg FASs. Northern California egg FASs had less overlap with CCV egg FASs, indicating a more regionally distinct diet. Eggs with FASs that indicated a northern anchovy/krill-dominated diet were also predominantly thiamine deficient, which indicates poor nutritional quality. Conversely, eggs with FASs that indicated a Pacific herring-dominated diet had higher total thiamine concentrations. Furthermore, most CCV Chinook salmon eggs were richer in lipids than most of those from NC, which likely indicates a greater northern anchovy contribution to the diet of CCV Chinook salmon.

#### Late fall-run

The Coleman National Fish Hatchery is the only late fall-run Chinook salmon hatchery located in the CCV, making it the sole sampling location for this Chinook salmon run (Table 3). About 40% of late fall-run Chinook salmon eggs sampled were affected by TDC (Table 4). Mean egg lipid content was 10.99 ± 0.82% (Table S5).

Most late fall-run Chinook salmon egg FASs were rich in EPA and DHA, but some eggs were rich in oleic acid (Table S5; Fig. S1C). Eggs with elevated concentrations of oleic acid also had elevated total thiamine concentrations (Fig. S1D). Eggs rich in EPA and DHA tended to have lower total thiamine concentrations.

The diet of late fall-run Chinook salmon appeared to consist mainly of northern anchovy or krill, as indicated by increased concentrations of EPA in their eggs. However, eggs with greater concentrations of oleic acid also had greater total thiamine reserves, corresponding to a diet of Pacific herring. Fewer eggs were rich in DHA, which indicates a lesser importance of market squid or rockfish in the diet.

#### Winter-run

Winter-run Chinook salmon were collected only from the Livingston Stone National Fish Hatchery, the sole winter-run hatchery located in CCV (Table 3). About 84% of winter-run Chinook salmon eggs sampled were affected by TDC (Table 4). Winter-run Chinook salmon eggs had the lowest total thiamine concentration among all Chinook salmon runs. Winter-run mean egg lipid content was the second highest, trailing CCV spring-run eggs (Table S5).

Winter-run Chinook salmon egg FASs had some of the highest proportions of EPA compared to other Chinook salmon runs (Table S5; Fig. S2A). Winter-run Chinook salmon eggs, to a lesser extent, were also rich in DHA. Eggs with greater DHA proportions had higher total thiamine concentrations, whereas eggs rich in EPA generally corresponded to females with critically low egg total thiamine concentrations (Fig. S2B).

Most winter-run Chinook salmon eggs had high EPA proportions and were thiamine deficient, which indicates a diet rich in northern anchovy or krill. The fewer number of eggs rich in DHA indicates the lesser importance of market squid or rockfish in the diet.

#### Spring-run

Spring-run Chinook salmon eggs were collected from one hatchery in CCV (Feather River Hatchery) and one hatchery in NC (Trinity River Hatchery; Table 3). Of all spring-run Chinook salmon eggs sampled in the CCV, most were unlikely to be impacted by TDC (Table 4). In NC, spring-run Chinook salmon egg total thiamine concentrations were even higher than those from CCV (Table 4). Also, CCV spring-run Chinook salmon eggs had a greater lipid content than NC spring-run Chinook salmon eggs (MW: *U* = 7471, *P* < 0.001; Tables S3 and S5).

Spring-run Chinook salmon egg FASs were significantly different between regions (PERMANOVA: Pseudo-F = 63.43, *df* = 1, *P* < 0.001; Tables S3 and S5). NC eggs were mostly rich in oleic acid, meanwhile CCV eggs were mostly rich in EPA (Fig. S2C). Eggs rich in oleic acid also had greater total thiamine reserves and eggs rich in EPA had lower total thiamine concentrations (Fig. S2D). A canonical analysis of principal coordinates indicated egg FASs were distinct, and therefore diets were distinct, between regions with 90% (NC) and 82% (CCV) of eggs correctly classified to their hatchery of origin.

Comparison of egg and prey FAS indicated NC spring-run Chinook salmon had a greater reliance on Pacific herring (elevated oleic acid proportion) in their diet, which was associated with higher egg total thiamine concentrations. Spring-run Chinook salmon eggs from CCV were rich in EPA with decreased egg total thiamine concentrations, indicating these Chinook salmon consumed more northern anchovy or krill.

#### Correlation of UI and FA proportions with egg total thiamine concentrations

**Figure 6:**
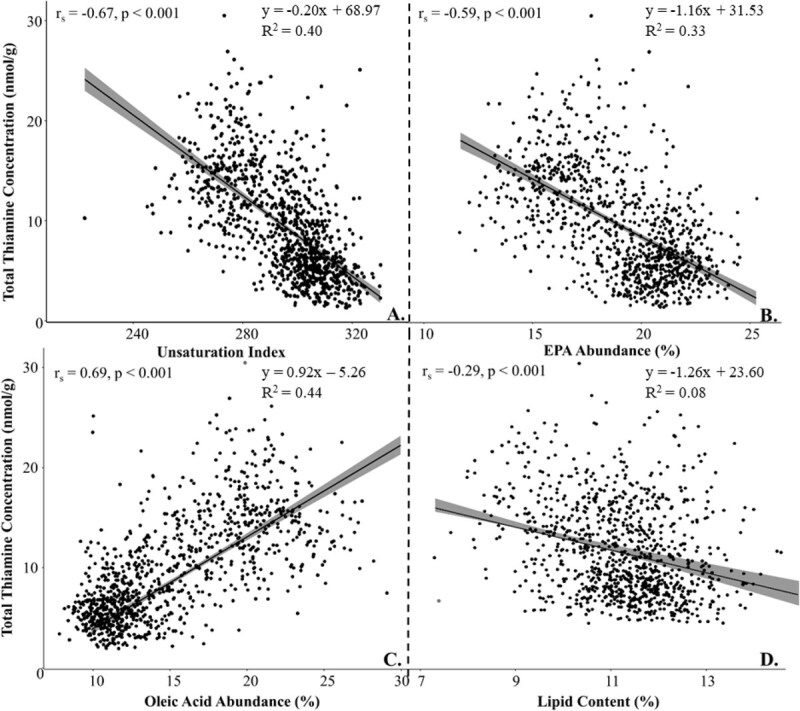
Spearman’s rank correlation between egg UI (**A**), EPA (**B**), oleic acid proportions (**C**) and lipid content (**D**) and total egg thiamine concentration of all Chinook salmon runs collected in 2020–2022. The grey-shaded area represents the 95% confidence interval (linear formula and *R*^2^ value are provided for each line). The Spearman’s rank correlation coefficient (*r_s_*) and *P*-value estimate (*P*) are provided for each plot.

To further address the nutritional quality of the Chinook salmon diet, egg UI and FA proportions were correlated with egg total thiamine concentration. Winter-run Chinook salmon had the highest mean egg UI (315.3 ± 7.0) and spring-run Chinook salmon had the lowest mean egg UI (285.3 ± 15.6). After pooling all egg UI from all Chinook salmon runs, UI had a significant negative correlation with egg free thiamine and total thiamine concentration (Table 5; Fig. 6A). Egg EPA proportions were negatively correlated with egg free thiamine and total thiamine concentrations (Fig. 6B). Conversely, egg oleic acid concentrations had a strong positive correlation with egg free thiamine and total thiamine concentrations (Fig. 6C). Lastly, egg lipid content had a weak negative correlation with egg total thiamine concentration and was significantly different among all runs except between fall-run and late fall-run (KW: *H* = 88.58, *df* = 3, *P* < 0.001; Fig. 6D).

**Table 5:** Spearman’s rank correlation (*r_s_*) values of Chinook salmon (CH; *O. tshawytscha*; *N* = 990), coho salmon (CO; *O. kisutch*; *N* = 186) and steelhead (STH; *O. mykiss*; *N* = 185) egg UI, fatty acids and egg lipid content with egg thiamine vitamers (TPP; thiamine pyrophosphate, TMP; thiamine monophosphate and TH; free thiamine) and egg total thiamine concentration

**Variables**	**Species**	**TPP**	**TMP**	**TH**	**Total thiamine**
Unsaturation index (UI)	CH	−0.019	−0.265^*^	−0.681^*^	−0.666^*^
	CO	−0.291^*^	−0.308^*^	−0.464^*^	−0.451^*^
	STH	−0.011	−0.222^*^	−0.486^*^	−0.499^*^
Eicosapentaenoic acid (%; EPA)	CH	0.184^*^	−0.168^*^	−0.628^*^	−0.591^*^
	CO	0.224^*^	−0.425^*^	−0.620^*^	−0.449^*^
	STH	0.024	−0.022	0.046	0.040
Docosahexaenoic acid (%; DHA)	CH	−0.245^*^	−0.112^*^	−0.177^*^	−0.199^*^
	CO	−0.044	0.233^*^	0.087	0.079
	STH	−0.053	−0.002	−0.285^*^	−0.286^*^
Oleic acid (%)	CH	−0.091^*^	0.184^*^	0.726^*^	0.695^*^
	CO	0.455^*^	0.471^*^	0.633^*^	0.635^*^
	STH	−0.100	0.371^*^	0.508^*^	0.512^*^
Lipid content (%)	CH	−0.169^*^	−0.213^*^	−0.275^*^	−0.294^*^
	CO	0.002	0.144	−0.139	−0.119
	STH	0.057	−0.027	−0.067	−0.040

^*^Significant correlation (*P* < 0.05).

### Thiamine concentrations, lipid contents and fatty acid proportions of *Coho Salmon* eggs

Coho salmon eggs were collected from the Trinity River and Iron Gate hatcheries, both located in NC (Table 6). Egg total thiamine concentrations were similar between both hatcheries. About 52% of eggs were unlikely impacted, 17% were likely to be impacted, and 31% were impacted by TDC (Table 4). Egg lipid content was significantly lower in Trinity River coho eggs compared to Iron Gate coho eggs (MW: *U* = 5136, *P* = 0.027; Table S6).

**Table 6:** Mean ± standard deviation of coho salmon (*O. kisutch*) and steelhead (*O. mykiss*) egg total thiamine concentrations (nmol/g) for unfertilized eggs collected during 2020–2022

**Species**	**Year**	**California’s Central Valley (CCV)**												**Northern California (NC)**			
		***N***	**Coleman**	***N***	**Feather**	***N***	**Nimbus**	***N***	**Mokelumne**	***N***	**Merced**	***N***	**Livingston**	***N***	**Trinity**	***N***	**Iron gate**
Coho	2020	0		0		0		0		0		0		31	15.25 ± 3.41	35	18.17 ± 4.89
	2021	0		0		0		0		0		0		30	7.46 ± 2.03	30	8.04 ± 3.77
	2022	0		0		0		0		0		0		30	5.09 ± 2.63	30	4.86 ± 2.90
Steelhead	2021	0		0		15	6.04 ± 2.53	13	14.08 ± 4.04	0		0		30	11.08 ± 3.45	0	
	2022	30	8.66 ± 3.08	30	11.09 ± 3.33	30	5.46 ± 2.01	7	10.41 ± 4.01	0		0		30	10.86 ± 3.41	0	

Sample size (*N*) is provided for each species per hatchery and year. A sample size of ‘0’ for all years indicates that species/run is not received at the given hatchery.

Coho salmon egg FASs were significantly different between Trinity River and Iron Gate hatcheries (PERMANOVA: Pseudo-F = 6.73, *df* = 1, *P* < 0.001; Table S6). Coho salmon from both hatcheries had eggs predominantly rich in EPA or oleic acid (Fig. S3A). Therefore, the ratio of EPA: oleic acid was able to delineate differences in diet (Fig. 5). Eggs that were richest in EPA (northern anchovy or krill diet) tended to have low total thiamine concentrations and those rich in oleic acid (Pacific herring diet) had high total thiamine concentrations (Fig. S3B). Results from a canonical analysis of principal coordinates showed that ~68 and 61% of individuals were correctly classified for the Trinity River and Iron Gate hatcheries, respectively.

**Figure 7:**
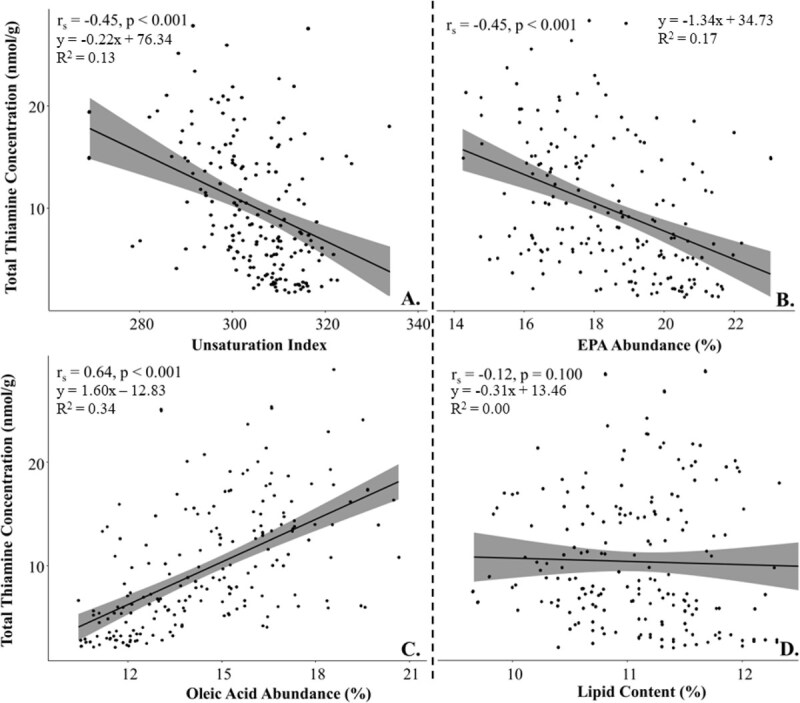
Spearman’s rank correlation between egg UI (A), EPA (B), oleic acid proportions (C) and lipid content (D) and total egg thiamine concentration of coho salmon (*O. kisutch*) collected from Trinity River and Iron Gate hatcheries in 2020–2022. The grey-shaded area represents the 95% confidence interval (linear formula and *R*^2^ value are provided for each line). The Spearman’s rank correlation coefficient (*r_s_*) and significance value (*P*) are provided for each plot.

Differences in the nutritional quality of the diet between Trinity River and Iron Gate coho salmon were found. Trinity River coho salmon eggs had a significantly greater mean UI than eggs from Iron Gate (306.6 ± 10.9 vs. 303.2 ± 9.8; MW: *U* = 3585, *P* = 0.045). After pooling the egg UI from both hatcheries, egg UI was negatively correlated with all thiamine vitamers and total thiamine concentration (Table 5; Fig. 7A). The egg EPA proportion was negatively correlated with TMP, TH and total thiamine (Table 5; Fig. 7B). All egg thiamine vitamers and total thiamine were positively correlated with the egg oleic acid proportion (Table 5; Fig. 7C). Lastly, egg lipid content was not strongly correlated with any thiamine vitamer or total thiamine concentration (Table 5; Fig. 7D).

### Thiamine concentrations, lipid contents and fatty acid proportions of steelhead eggs

Steelhead eggs were collected from the Mokelumne, Nimbus, Coleman and Feather River hatcheries in CCV and the Trinity River Hatchery in NC (Table 6). Of all steelhead eggs sampled in CCV, 51% were unlikely to be impacted by TDC (Table 4). However, 24% of CCV steelhead eggs were severely impacted, 13% were impacted, and 12% were likely to be impacted by TDC. Egg samples from the Nimbus River Hatchery contributed 78% of all eggs with low total thiamine concentrations (severely impacted or impacted). Of the steelhead eggs sampled in NC, 82% had high total thiamine concentrations (Table 4). CCV steelhead egg lipid content was not significantly different from that of NC’s (MW: *U* = 3384, *P* = 0.284; Table S7).

Steelhead egg FASs were significantly different across all hatcheries except for those collected at the Feather River and Coleman hatcheries (PERMANOVA: Pseudo-F = 66.643, *df* = 4, *P* < 0.001; Table S7). Eggs collected from the Nimbus Hatchery were generally rich in DHA (Fig. S3C). Eggs collected from the Coleman, Mokelumne and Feather River hatcheries were rich in EPA. Lastly, there was variation in the FASs of Trinity River Hatchery among individuals, as some eggs were rich in EPA and others were rich in oleic acid or DHA. Unlike Chinook and coho salmon, low egg total thiamine concentrations were largely related to high DHA and low oleic acid proportions (Fig. 5). Eggs rich in DHA were severely impacted by thiamine deficiency (Fig. S3D). A canonical analysis of principal coordinates showed that the Nimbus (100%) and Trinity River (72%) hatcheries had the highest percentage of correctly classified eggs to the hatchery at which they were collected based on FAS, whereas the Feather River, Mokelumne and Coleman hatcheries had ~50% of steelhead correctly identified by FAS.

Nimbus Hatchery eggs were impacted the most by TDC and had the highest proportions of DHA compared to other steelhead hatcheries. Eggs rich in DHA indicated a potential diet of market squid or rockfish. However, some eggs were rich in EPA, which indicated that females fed on northern anchovy or krill.

**Figure 8:**
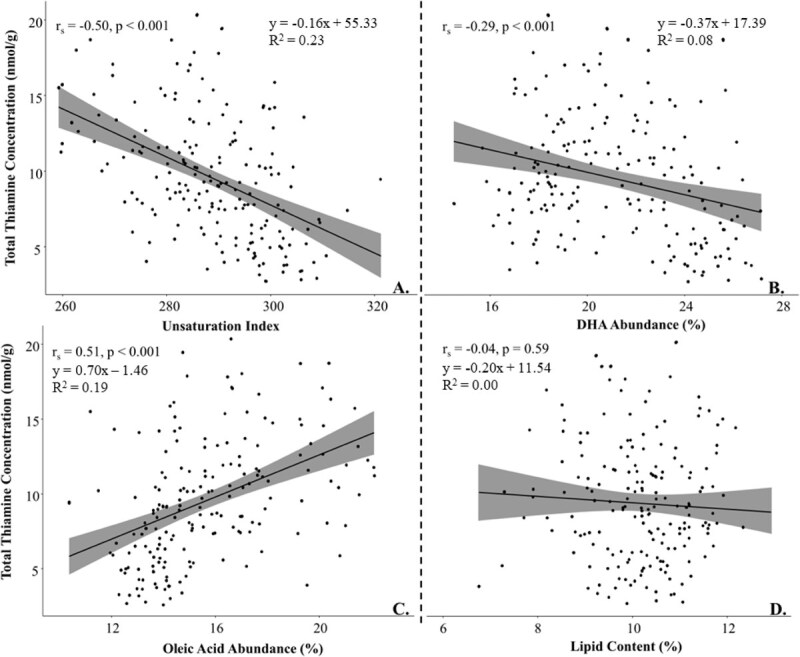
Spearman’s rank correlation between egg UI (**A**), DHA (**B**), oleic acid proportions (**C**) and lipid content (**D**) and total egg thiamine concentration of steelhead (*O. mykiss*) collected from California Central Valley (CCV) and Northern California (NC) hatcheries in 2020–2022. The grey-shaded area represents the 95% confidence interval (linear formula and *R*^2^ value are provided for each line). The Spearman’s rank correlation coefficient (*r_s_*) and significance value (*P*) are provided for each plot.

When comparing the nutritional quality of the diet, Nimbus Hatchery steelhead eggs had the highest mean UI, whereas Trinity River eggs had the lowest (301.1 ± 6.3 vs. 282.8 ± 11.1; KW: *H* = 71.80, *df* = 4, *P* < 0.001). Egg UI was pooled and found to be negatively correlated with free thiamine and total thiamine (Table 5; Fig. 8A). The egg EPA proportion was not significantly correlated with egg thiamine vitamers or total thiamine. The egg DHA proportion was negatively correlated with free thiamine and total thiamine (Table 5; Fig. 8B). Egg total thiamine was positively correlated with the oleic acid proportion (Fig. 8C). Lastly, steelhead egg lipid content was not significantly correlated with any thiamine vitamer or total thiamine concentration (Table 5; Fig. 8D).

### Fatty acid signature analysis of all salmonine species

**Figure 9:**
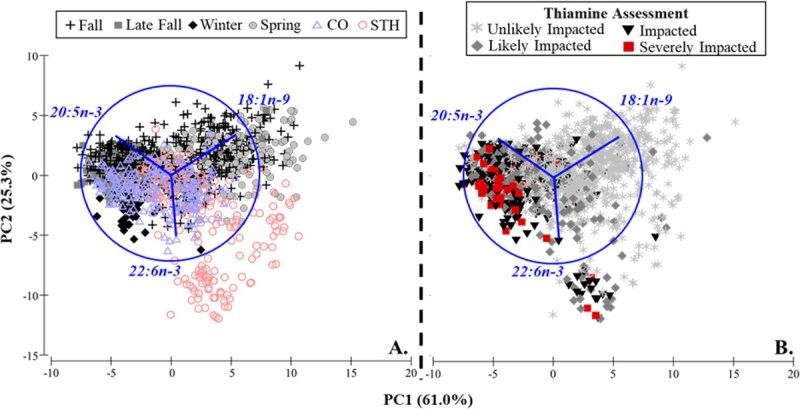
(**A**) PCA of all Chinook salmon (*O. tshawytscha*) runs (fall, late fall, winter, spring), coho salmon (*O. kisutch*; CO) and steelhead (*O. mykiss*; STH) egg FAS based on the proportions of fatty acids. Vectors for 18:1n-9, 20:5n-3 and 22:6n-3 are included based on their loading values. The percent variation accounted for by each PC is included in parentheses on the axis title (PCs are the same for plots A and B). (**B**) Egg FASs are plotted with their corresponding egg thiamine concentrations.

Differences in salmonine egg FAS indicated differences in diet and/or in the proportions of each prey species contained in the diet among each salmonine species. Egg FASs were significantly different among all salmonine runs/species (Chinook fall-run, Chinook late fall-run, Chinook winter-run, Chinook spring-run, coho salmon and steelhead; PERMANOVA: Pseudo-F = 138.66, *df* = 5, *P* < 0.001; Fig. 9A). Eggs collected from fall-run Chinook salmon had a large variation in FAS, particularly EPA (CCV: *X̅* = 19.95%, NC: *X̅* = 17.83%) and oleic acid (CCV: *X̅* = 14.86%, NC: *X̅* = 18.70%) proportion. Eggs from CCV fall-run and late fall-run Chinook salmon were rich in EPA (*X̅* = 19.95% and *X̅* = 20.86%, respectively). Similarly, winter-run Chinook salmon and coho salmon eggs had greater proportions of EPA (*X̅* = 20.37% and *X̅* = 18.41%, respectively) and DHA (*X̅* = 20.53% and *X̅* = 19.52%, respectively). Spring-run and NC fall-run Chinook salmon eggs were rich in oleic acid (*X̅* = 19.61% and *X̅* = 18.70%, respectively). The lowest egg FAS similarity seen within a run or species was in steelhead eggs (SIMPER: mean similarity = 90.1%), demonstrating diversity in their diet. Nimbus River Hatchery steelhead eggs were rich in DHA (*X̅* = 21.12%), proportions larger than that of any other steelhead population or species; these eggs were also thiamine deficient. In contrast, eggs rich in EPA had the greatest presence of thiamine deficiency among Chinook and coho salmon (Fig. 9B).

## Discussion

Salmonine egg FASs revealed both intra- and interpopulation differences in dietary patterns. Variations in egg FASs, largely driven by differences in oleic acid, EPA and DHA proportions, were observed across regions, hatcheries and populations/species. The main prey species characterized by these fatty acids were Pacific herring (oleic acid), northern anchovy and krill (EPA), and market squid and rockfish (DHA). Our results suggest that these three fatty acids, coupled with prey nutritional content, can serve as effective biomarkers for the diets of Chinook salmon, coho salmon and steelhead.

Differences in prey consumption were consistent with diet-induced differences in salmonine egg total thiamine concentrations at spawning (Mantua *et al*., 2025). The first objective of diet determination was addressed because eggs with elevated EPA proportions suggested that northern anchovy or krill were major dietary components. Eggs with elevated oleic acid proportions suggested that Pacific herring was a large contributor to the diet. Regarding the second objective, eggs rich in EPA tended to have lower total thiamine concentrations. However, a krill-dominated diet seems unlikely to cause low egg total thiamine concentrations because krill are high in total thiamine, low in lipids and have low thiaminase activity. Although not measured, krill are also rich in the antioxidant astaxanthin, which may confer additional health benefits (Bell *et al*., 2000). In contrast, an anchovy-dominated diet is more likely to drive a low egg total thiamine concentration in California salmonine species because northern anchovies on average had the highest thiaminase activity, the highest lipid content (as well as high PUFA content) and the lowest total thiamine concentration among the prey species examined. These findings are consistent with the gut content analysis reported by Mantua *et al*. (2025) that showed anchovy-dominated Chinook salmon diets in years when thiamine deficiency was widespread in Central Valley Chinook salmon egg samples. Moreover, stable isotope analysis of coho and Chinook salmon eggs in that study also identified a strong negative correlation between total thiamine concentration and δ^15^N, indicating that thiamine-deficient salmon were feeding at higher trophic levels. It is likely that similarities in EPA proportions between northern anchovy and krill reflect that northern anchovy prey heavily on krill, particularly during early life stages, when they overlap in space and time; as anchovy are known to selectively feed on larger planktonic prey (Koslow, 1981; Rykaczewski and Checkley Jr, 2008).

Patterns in egg FAS and associated prey also provided insight on prey quality. Eggs rich in oleic acid suggested primary consumption of Pacific herring, and these eggs generally had relatively high total thiamine concentrations. Herring are the second most lipid-rich prey considered, have relatively high thiaminase activity (trailing only anchovy), and have an intermediate total thiamine content. This may suggest that lipid quality may be more important than lipid quantity, as the proportion of PUFAs in herring is less than in anchovy. Also, these results may indicate that there is a threshold as to when thiaminase activity/PUFA proportions become detrimental and cause thiamine deficiency. Nimbus Hatchery steelhead eggs were thiamine deficient, but egg FASs (rich in DHA) suggest a diet of market squid or juvenile rockfish. However, this prey exhibited high total thiamine concentrations, low lipid contents and low thiaminase activities, all of which are not conducive to causing thiamine deficiency. Thiamine deficiency in this population could stem from the presence of PUFAs which may be peroxidized, or since Nimbus Hatchery steelhead generally undergo long offshore migrations, they may potentially encounter different prey compared to the coho and Chinook salmon populations investigated here. Variations in egg DHA proportions are also potentially connected with variations in market squid or juvenile rockfish populations along the West Coast, which are both subject to high interannual variability in abundance (Ralston *et al*., 2013; Thompson *et al*., 2019).

The availability and relative abundance of key salmon prey appear to be highly variable in both space and time. Northern anchovy were more abundant in central/southern California in recent years, while Pacific herring were more abundant off the coast of Northern California, Oregon and Washington (Thompson *et al*., 2017; Stierhoff *et al*., 2024; Hinchliffe *et al*., 2025). This may be a relatively recent phenomena, as biomass estimates of the primary central California herring population (San Francisco Bay) suggest that the biomass in 2020 was <15% of the long-term (1979–2000) value, and <5% of the peak values in that period (California Department of Fish and Wildlife, unpublished data). Conversely, the extremely high recent biomass estimates of northern anchovy are limited to the region south of Cape Mendocino, which is a separate stock (the Central Northern Subpopulation), while northern anchovy biomass levels north of Cape Mendocino (the Northern Subpopulation) have been orders of magnitude lower (Litz *et al*., 2008; Stierhoff *et al*., 2024; Hinchcliffe *et al.*, 2025). Thus, areas associated with high juvenile rockfish and/or market squid abundance are likely associated with less reliance on anchovy and other clupeid forage species (and thus higher DHA proportions), while areas with low abundance are likely associated with greater predation on anchovy (thus lower DHA and higher EPA proportions). Such a prey-switching dynamic has been well documented in seabirds in the Central California Current ecosystem that rely on the same suite of forage taxa during late spring and early summer (Wells *et al*., 2017). Consequently, the low pelagic juvenile rockfish abundance in the 2020–2022 period (Thompson *et al*., 2024), coupled with the precipitous decline of Pacific herring in Central California waters and the highest northern anchovy abundance observed since the early 1970s are all consistent with anchovy-dominated diets for salmon foraging primarily in central California. Salmonines foraging north of Cape Mendocino, in northern California and southern Oregon marine waters, had a diet not dominated by anchovies, as indicated by our results.

These observations would support the hypothesis that within regional populations for each species, annual variations in egg total thiamine concentrations were generally synchronous across hatcheries. This suggests that regional populations likely visit similar feeding areas and have access to similar prey species (Satterthwaite *et al*., 2013; Satterthwaite *et al*., 2015).

Additional research to understand the causes behind variations in egg FAS and diet, especially in relation to steelhead from Nimbus River Hatchery, could be beneficial. Differences in diet may be influenced by migration patterns, with some steelhead stocks undertaking short, coastal migrations while others embark on longer offshore routes, thereby accessing different prey species (Teo *et al*., 2013; Myers, 2018). Future work is needed to investigate how variation in lipid class composition (e.g. triacylglycerols and phospholipids) influences the overall FAS and its biological significance. Furthermore, the metabolism and role in vitellogenesis of specific fatty acids (oleic acid, EPA, DHA) should be considered as these fatty acids can be selectively transferred from the mother to her eggs and ultimately influence the egg FAS.

Beyond egg FAS, egg quality also varied. Egg quality was assessed by lipid content and fatty acid proportions and their correlations with thiamine vitamers and total thiamine to understand the potential impacts of dietary shifts. While lipid and fatty acid correlations with total thiamine and free thiamine were similar, the correlations were generally stronger with total thiamine. Across all salmonine species, egg lipid content was negatively correlated with total thiamine concentrations, but only significantly for Chinook salmon (all runs pooled). This is consistent with results from Baltic Atlantic salmon suggesting that lipid content from other tissues (i.e. muscle) rather than eggs is more indicative of thiamine status (Vuorinen *et al*., 2024). However, egg UI was negatively correlated with total thiamine concentrations for all species, with the highest UI values (and thus the highest PUFA proportions) observed in the most thiamine-deficient eggs. These findings support the hypothesis that excessive PUFA intake contributes to thiamine deficiency due to the greater risk of oxidative stress (Keinänen *et al*., 2018; Keinänen *et al*., 2022).

In conclusion, our findings highlight that a shifting prey base has tremendous top-level influences on the food web. Maternal diet was determined using fatty acid analysis, indicating the dominance of northern anchovy in most diets. With the northern anchovy being a prey species with high thiaminase activity, high lipid and PUFA content, and low total thiamine concentrations, egg quality and offspring survival are negatively impacted. Since the UI of eggs (which reflects the number of double bonds and thus the peroxidation potential of PUFAs) had the strongest negative correlation with egg total thiamine concentration, the observed thiamine deficiency is likely linked to high proportions of PUFAs in the diet as well as thiaminase consumption, both of which consume or degrade thiamine. Our results support the hypotheses that diet-induced thiamine deficiency is associated with a diet dominated by prey having high thiaminase activity in California’s Chinook salmon, coho salmon, and most steelhead and is also associated with lipid quality as tracked by the UI. Additional work needs to be done to explore the interactions of these two thiamine deficiency pathways.

The presence of TDC in threatened or federally protected populations of salmonines represents an additional hindrance towards recovery. Meanwhile, the emergence of TDC in harvest stocks (fall-run Chinook salmon) may limit recruitment and cause population downturns. However, the early detection of TDC in these salmonine populations allows for mitigation, especially for populations where broodstock is collected for propagation (like those studied here). Egg thiamine baths or prenatal thiamine injections prior to spawning have shown promising results in several systems for recovering ailing offspring from symptoms of TDC (Futia *et al*., 2017; Mantua *et al*., 2025). From a conservation perspective, thiamine treatments must be deeply considered as they may provide individual fitness benefits but cause evolutionary disadvantages through the survival of individuals intolerant to low thiamine environments (Harder *et al*., 2024).

## Supplementary Material

Web_Material_coag037

## Data Availability

Data will be made available upon reasonable request.

## References

[ref1] Alvarez MJ, Lopez-Bote CJ, Diez A, Corraze G, Arzel J, Dias J, Kaushik SJ, Bautista JM (1998) Dietary fish oil and digestible protein modify susceptibility to lipid peroxidation in the muscle of rainbow trout (*Oncorhynchus mykiss*) and sea bass (*Dicentrarchus labrax*). Br J Nutr 80: 281–289. 10.1017/S0007114598001330. 9875068

[ref2] Balon EK (1975) Terminology of intervals in fish development. J Fish Res Board Can 32: 1663–1670. 10.1139/f75-196.

[ref3] Bell HN (2022) Assessment of the effects of thiamine deficiency on the survival, physiology, and behavior of early life-stage winter-run Chinook salmon. Dissertation. University of California-Davis, California.

[ref4] Bell JG, McEvoy J, Tocher DR, Sargent JR (2000) Depletion of α-tocopherol and astaxanthin in Atlantic Salmon (*Salmo salar*) affects autoxidative defense and fatty acid metabolism. J Nutr 130: 1800–1808. 10.1093/jn/130.7.1800. 10867054

[ref7] Brown SB, Honeyfield DC, Vandenbyllaardt L (1998) Thiamine analysis in fish tissues. In G McDonald, JD Fitzsimons, DC Honeyfield, eds, Early Life Stage Mortality Syndrome in Fishes of the Great Lakes and Baltic Sea. American Fisheries Society, Bethesda, MD, pp. 73–81

[ref8] Chen J, Liu H (2020) Nutritional indices for assessing fatty acids: a mini-review. Int J Mol Sci 21: 5695. 10.3390/ijms21165695. 32784511 PMC7460856

[ref9] Dalsgaard J, John M, Kattner G, Muller-Navarra D, Hagen W (2003) Fatty acid trophic markers in pelagic marine environment. Adv Mar Biol 46: 225–340. 10.1016/S0065-2881(03)46005-7. 14601414

[ref10] Daly EA, Benkwitt CE, Brodeur RD, Litz MNC, Copeman LA (2010) Fatty acid profiles of juvenile salmon indicate prey selection strategies in coastal marine waters. Mar Biol 157: 1975–1987. 10.1007/s00227-010-1466-9.

[ref11] Fernandez-Palacios H, Soledad Izquierdo M, Robaina L, Valencia A, Salhi M, Vergara JM (1995) Effect of *n-3* HUFA level in broodstock diets on egg quality of gilthead sea bream (*Sparus aurata* L.). Aquac 132: 325–337. 10.1016/0044-8486(94)00345-O.

[ref12] Field JC, Miller RR, Santora JA, Tolimieri N, Haltuch MA, Brodeur RD, Auth TD, Dick EJ, Monk MH, Sakuma KM et al. (2021) Spatiotemporal patterns of variability in the abundance and distribution of winter-spawned pelagic juvenile rockfish in the California Current. PloS One 16: e0251638. 10.1371/journal.pone.0251638. 34043656 PMC8158922

[ref13] Fitzsimons JD, Brown SB (1998) Reduced egg thiamine levels in inland and Great Lakes lake trout and their relationship with diet. In G McDonald, JD Fitzsimons, DC Honeyfield, eds, Early Life Stage Mortality Syndrome in Fishes of the Great Lakes and Baltic Sea. American Fisheries Society, Bethesda, MD, pp. 160–171.

[ref14] Folch J, Lees M, Sloane-Stanely G (1957) A simple method for the isolation and purification of total lipids from animal tissues. J Biol Chem 226: 497–509. 10.1016/S0021-9258(18)64849-5. 13428781

[ref15] Futia MH, Hallenbeck S, Noyes AD, Honeyfield DC, Eckerlin GE, Rinchard J (2017) Thiamine deficiency and the effectiveness of thiamine treatments through broodstock injections and egg immersion on Lake Ontario steelhead trout. J Great Lakes Res 43: 352–358. 10.1016/j.jglr.2017.01.001.

[ref16] Futia MH, Rinchard J (2019) Evaluation of adult and offspring thiamine deficiency in salmonine species from Lake Ontario. J Great Lakes Res 45: 811–820. 10.1016/j.jglr.2019.05.010.

[ref5] Green, RG (1936) Chastek paralysis - a new disease of foxes. Minnesota Wildlife Disease Investigation 2: 106–107.

[ref17] Greig RA, Gnaedinger RH (1971) Occurrence of thiaminase in some common aquatic animals of the United States and Canada. NOAA and NMFS Special Sci Rep Fish 631: 1–7.

[ref18] Groot C, Margolis L (1991) Pacific Salmon Life Histories. *In* C Groot and L Margolis. UBC Press, Vancouver, BC.

[ref19] Halver JE (1972) The vitamins. In Fish Nutrition. Academic Press, San Diego, CA.

[ref20] Hanes JW, Kraft CE, Begley TP (2007) An assay for thiaminase I in complex biological samples. Analyt Biochem 368: 33–38. 10.1016/j.ab.2007.06.001. 17603991 PMC2140240

[ref21] Happel A, Pattridge R, Walsh M, Rinchard J (2017) Assessing diet compositions of Lake Ontario predators using fatty acid profiles of prey fishes. J Great Lakes Res 43: 838–845. 10.1016/j.jglr.2016.12.008.

[ref22] Harder AM, Reed AN, Rowland F (2024) Evolutionary perspectives on thiamine supplementation of managed Pacific salmonid populations. Can J Fish Aquat Sci 82: 1–10. 10.1139/cjfas-2024-0109.

[ref23] Hayes SA, Ammann AJ, Harding JA, Hassrick JL, deWitt L, Morgan CA (2016) Observations of steelhead in the California Current lead to a marine-based hypothesis for the “half-pounder” life history, with climate change implications for anadromy. N Pac Anadr Fish Comm Bull 6: 97–105. 10.23849/npafcb6/97.105.

[ref24] Hinchliffe C, Kuriyama PT, Punt AE, Field JC, Thompson AR, Santora JA, Muhling BA, Koenigstein S, Hernvann PY, Tommasi D (2025) Long-term population trend of northern anchovy (*Engraulis mordax*) in the California Current system. ICES J Mar Sci 82: fsae177. 10.1093/icesjms/fsae177.

[ref25] Honeyfield DC, Hinterkopf JP, Fitzsimons JD, Tillitt DE, Zajicek JL, Brown SB (2005) Development of thiamine deficiencies and early mortality syndrome in lake trout by feeding experimental and feral fish diets containing thiaminase. J Aquat Anim Health 17: 4–12. 10.1577/H03-078.1.

[ref26] Izquierdo MS, Fernández-Palacios H, Tacon AGJ (2001) Effect of broodstock nutrition on reproductive performance of fish. Aquaculture 197: 25–42. 10.1016/S0044-8486(01)00581-6.

[ref27] Keinänen M, Käkelä R, Ritvanen T, Pönni J, Harjunpää H, Myllylä T, Vuorinen PJ (2018) Fatty acid signatures connect thiamine deficiency with the diet of the Atlantic salmon (*Salmo salar*) feeding in the Baltic Sea. Mar Biol 165: 161. 10.1007/s00227-018-3418-8. 30369636 PMC6182616

[ref28] Keinänen M, Nikonen S, Käkelä R, Ritvanen T, Rokka M, Myllylä T, Pönni J, Vuorinen PJ (2022) High lipid content of prey fish and n−3 PUFA peroxidation impair the thiamine status of feeding-migrating Atlantic salmon (*Salmo salar*) and is reflected in hepatic biochemical indices. Biomolecules 12: 526. 10.3390/biom12040526. 35454115 PMC9031544

[ref29] Keinänen M, Raitaniemi J, Pönni J, Ritvanen T, Myllylä T, Vuorinen P (2025) Reduced numbers of returning Atlantic salmon (*Salmo salar*) and thiamine deficiency are both associated with the consumption of high-lipid prey fish. Fishes 10: 10010016.

[ref30] Keinänen M, Uddström A, Mikkonen J, Pönni J, Myllylä T, Aro E, Vuorinen PJ (2012) The thiamine deficiency syndrome M74, a reproductive disorder of Atlantic salmon (*Salmo salar*) feeding in the Baltic Sea, is related to the fat and thiamine content of prey fish. ICES J Mar Sci 69: 516–528. 10.1093/icesjms/fss041.

[ref31] Koslow AJ (1981) Feeding selectivity of schools of northern anchovy, *Engraulis mordax*, in the Southern California bight. Fish Bull 79: 131–142.

[ref32] Kraft CE, Gordon ERL, Angert ER (2014) A rapid method for assaying thiaminase I activity in diverse biological samples. PloS One 9: 92688. 10.1371/journal.pone.0092688. PMC3968017 24675843

[ref33] Lehninger AL (1970) Biochemistry: The Molecular Basis of Cell Structure And Function. Worth Publishers, New York, NY, pp. 129–138.

[ref34] Litz MN, Heppell SS, Emmett RL, Brodeur RD (2008) Ecology and distribution of the northern subpopulation of northern anchovy (*Engraulis mordax*) off the US west coast. California Coop Ocean Fish Investig Reps 49: 167–182.

[ref35] Lovern JA (1935) Fat metabolism in fishes. The fats of some plankton crustacea. Biochem J 29: 847–849. 10.1042/bj0290847. 16745734 PMC1266560

[ref36] Ludwig JM (2024) Assessing diets of California salmonines using fatty acid signatures and its impact on observed thiamine deficiency. Master’s thesis. State University of New York–Brockport, Brockport, NY.

[ref38] MacCall AD (1996) Patterns of low-frequency variability in fish populations of the California Current. California Coop Ocean Fish Investig Reps 37: 100–110.

[ref39] Mantua N, Bell H, Daniels M, Rinchard J, Ludwig J, Tillitt D, Honeyfield D, Lipscomb T, Field J, Lindley S et al. (2025) Widespread thiamine deficiency in California salmon linked to a simplified anchovy-dominated marine prey base. Proc Natl Acad Sci 122: e2426011122. 10.1073/pnas.2426011122. 40549902 PMC12232615

[ref40] Mantua N, Johnson R, Field J, Lindley S, Williams T, Todgham A, Fangue N, Jeffres C, Bell H, Cocherell D et al. (2021) Mechanisms, impacts, and mitigation for thiamine deficiency and early life stage mortality in California’s Central Valley Chinook salmon. N Pac Anadr Fish Comm Tech Rep 17: 92–93. 10.23849/npafctr17/92.93.

[ref41] Merkel TJ (1957) Food habits of the king salmon, *Oncorhynchus tshawytscha* (Walbaum), in the vicinity of San Francisco, California. Calif Fish and Game 43: 249–270.

[ref42] Metcalfe L, Schmitz A (1961) The rapid preparation of fatty acid esters for gas chromatographic analysis. Anal Chem 33: 363–364. 10.1021/ac60171a016.

[ref43] Morito CLH, Conrad DH, Hilton JW (1986) The thiamine deficiency signs and requirement of rainbow trout (*Salmo gairdneri*, Richardson). Fish Physiol Biochem 1: 93–104. 10.1007/BF02290209. 24234598

[ref44] Myers K (2018) Ocean ecology of steelhead. In RJ Beamish, ed, The Ocean Ecology of Pacific Salmon and Trout. American Fisheries Society, Bethesda, MD, pp. 779–904.

[ref45] Napolitano GE (1999) Fatty acids as trophic and chemical markers in freshwater ecosystems. In MT Arts, BC Wainman, eds, Lipids in Freshwater Ecology. Springer, New York, NY, pp. 21–44

[ref46] Navas JM, Bruce M, Thrush M, Farndale BM, Bromage N, Zanuy S, Carrillo M, Bell JG, Ramos J (1997) The impact of seasonal alteration in the lipid composition of broodstock diets on egg quality in the European sea bass. J Fish Biol 51: 760–773. 10.1111/j.1095-8649.1997.tb01997.x.

[ref47] Pickova J, Kiessling A, Pettersson A, Dutta PC (1998) Comparison of fatty acid composition and astaxanthin content in healthy and by M74 affected salmon eggs from three Swedish river stocks. Comp Biochem Physiol Part B: Biochem Molec Biol 120: 265–271. 10.1016/S0305-0491(98)10016-0.

[ref48] Pickova J, Kiessling A, Pettersson A, Dutta PC (1999) Fatty acid and carotenoid composition of eggs from two nonanadromous Atlantic salmon stocks of cultured and wild origin. Fish Physiol Biochem 21: 147–156. 10.1023/A:1007860908911.

[ref49] Rainuzzo JR (1993) Fatty acid and lipid composition of fish egg and larvae. In H Reinersten, LA Dahle, L Jorgensen, eds, Fish Farming Technology. CRC Press, Boca Raton, FL, pp. 43–48.

[ref50] Ralston S, Sakuma KM, Field JC (2013) Interannual variation in pelagic juvenile rockfish (*Sebastes* spp.) abundance – going with the flow. Fish Oceano 22: 288–308. 10.1111/fog.12022.

[ref51] Richter CA, Evans AN, Heppell SA, Zajicek JL, Tillitt DE (2023) Genetic basis of thiaminase I activity in a vertebrate, zebrafish *Danio rerio*. Sci Rep 13: 698. 10.1038/s41598-023-27612-5. 36639393 PMC9839694

[ref52] Rodriguez C, Cejas RJ, Martin MV, Badía P, Samper M, Lorenzo A (1998) Influence of *n*–3 highly unsaturated fatty acid deficiency on the lipid composition of broodstock gilthead seabream (*Sparus aurata* L.) and on egg quality. Fish Physiol Biochem 18: 177–187. 10.1023/A:1007750218840.

[ref53] Rowland FE, Byrd CG, Kroboth PT (2026) Thiaminase I activity is high in grass and silver carp, but negligible in bighead and black carp. J Great Lakes Res 52: 102751. 10.1016/j.jglr.2026.102751.

[ref54] Rykaczewski RR, Checkley DM Jr (2008) Influence of ocean winds on the pelagic ecosystem in upwelling regions. Proc Natl Acad Sci 105: 1965–1970. 10.1073/pnas.0711777105. 18250305 PMC2538866

[ref55] Santora JA, Schroeder ID, Bograd SJ, Chavez FP, Cimino MA, Fiechter J, Hazen EL, Kavanaugh MT, Messié M, Miller RR et al. (2021) Pelagic biodiversity, ecosystem function, and services. Oceano 34: 16–37. 10.5670/oceanog.2021.212.

[ref56] Sargent J, Bell G, McEvoy L, Tocher D, Estevez A (1999) Recent developments in the essential fatty acid nutrition of fish. Aquaculture 177: 191–199. 10.1016/S0044-8486(99)00083-6.

[ref57] Satterthwaite WH, Ciancio J, Crandall E, Palmer-Zwahlen ML, Grover AM, O’Farrell MR, Anderson EC, Mohr MS, Garza JC (2015) Stock composition and ocean spatial distribution inference from California recreational Chinook salmon fisheries using genetic stock identification. Fish Res 170: 166–178. 10.1016/j.fishres.2015.06.001.

[ref58] Satterthwaite WH, Mohr MS, O’Farrell MR, Wells BK (2013) A comparison of temporal patterns in the ocean spatial distribution of California’s Central Valley Chinook salmon runs. Can J Fish Aquat Sci 70: 574–584. 10.1139/cjfas-2012-0395.

[ref59] Schwartzlose RA, Alheit J, Bakun A, Baumgartner TR, Cloete R, Crawford RJM, Fletcher WJ, Green-Ruiz Y, Hagen E, Kawasaki T et al. (1999) Worldwide large-scale fluctuations of sardine and anchovy populations. S Afr J Mar Sci 21: 289–347. 10.2989/025776199784125962.

[ref60] Stierhoff KL, Zwolinski JP, Renfree JS, Demer DA (2024) Distribution, biomass, and demographics of coastal pelagic fishes in the California Current Ecosystem during summer 2023 based on acoustic-trawl sampling. NOAA Tech Memo NMFS SWFSC 703: 1–82.

[ref61] Suca JJ, Santora JA, Field JC, Curtis KA, Muhling BA, Cimino MA, Hazen EL, Bograd SJ (2022) Temperature and upwelling dynamics drive market squid (*Doryteuthis opalescens*) distribution and abundance in the California Current. ICES J Mar Sci 79: 2489–2509. 10.1093/icesjms/fsac186.

[ref62] Szoboszlai AI, Thayer JA, Wood SA, Sydeman WJ, Koehn LE (2015) Forage species in predator diets: synthesis of data from the California Current. Ecolol Inform 29: 45–56. 10.1016/j.ecoinf.2015.07.003.

[ref63] Teo SLH, Sandstrom PT, Chapman ED, Null RE, Brown K, Klimley AP, Block BA (2013) Archival and acoustic tags reveal the post-spawning migrations, diving behavior, and thermal habitat of hatchery-origin Sacramento River steelhead kelts (*Oncorhynchus mykiss*). Env Biol Fish 96: 175–187. 10.1007/s10641-011-9938-4.

[ref64] Thayer JA, Field JC, Sydeman WJ (2014) Changes in California Chinook salmon diet over the past 50 years: relevance to the recent population crash. Mar Ecol Prog Ser 498: 249–261. 10.3354/meps10608.

[ref65] Thompson AR, Harvey CJ, Sydeman WJ, Barceló C, Bograd SJ, Brodeur RD, Fiechter J, Field JC, Garfield N, Good TP et al. (2019) Indicators of pelagic forage community shifts in the California Current Large Marine Ecosystem, 1998–2016. Ecol Indic 105: 215–228. 10.1016/j.ecolind.2019.05.057.

[ref66] Thompson AR, Swalethorp R, Alksne M, Santora JA, Hazen EL, Leising A, Satterthwaite WH, Sydeman WJ, Anderson CR, Auth TD et al. (2024) State of the California Current Ecosystem report in 2022: a tale of two La Niñas. Front Mar Sci 11: 1294011. 10.3389/fmars.2024.1294011.

[ref67] Thompson SA, Sydeman WJ, Thayer JA, Weinstein A, Krieger KL, Hay D (2017) Trends in the Pacific herring (*Clupea pallasii*) metapopulation in the California Current Ecosystem. California Coop Ocean Fish Investig Reps 58: 77–94.

[ref68] Vuorinen PJ, Käkelä R, Pakarinen T, Heinimaa P, Ritvanen T, Nikonen S, Rokka M, Keinänen M (2024) Thiamine deficiency M74 developed in salmon (*Salmo salar*) stocks in two Baltic Sea areas after the hatching of large year-classes of two clupeid species–detected by fatty acid signature analysis. Fishes 9: 1–35. 10.3390/fishes9020058.39380839

[ref69] Wells BK, Santora JA, Bizzarro JJ, Billings A, Brodeur RD, Daly EA, Field JC, Richerson KE, Thorson JT (2024) Trophoscapes of predatory fish reveal biogeographic structuring of spatial dietary overlap and inform fisheries bycatch patterns. Mar Ecol Prog Ser 741: 47–70. 10.3354/meps14319.

[ref70] Wells BK, Santora JA, Henderson MJ, Warzybok P, Jahncke J, Bradley RW, Huff DD, Schroeder ID, Nelson P, Field JC et al. (2017) Environmental conditions and prey-switching by a seabird predator impact juvenile salmon survival. J Mar Syst 174: 54–63. 10.1016/j.jmarsys.2017.05.008.

[ref6] Wolf, LE (1942) Fish-diet disease of trout: a vitamin deficiency produced by diets containing raw fish. Fisheries Research Bulletin No. 2. New York State Department of Environmental Conservation, Albany.

