# Supplementary material for: Fatty acid profiles recorded in ocean prey and California salmonine eggs reveal maternal ocean diets linked to thiamine deficiency
Source: Conserv Physiol. 2026 Jun 10;14(1):coag037. doi: 10.1093/conphys/coag037 (PMC13253152; doi:10.1093/conphys/coag037)
Supplement: Web_Material_coag037 [file web_material_coag037.zip › Supplemtary materials.pdf]

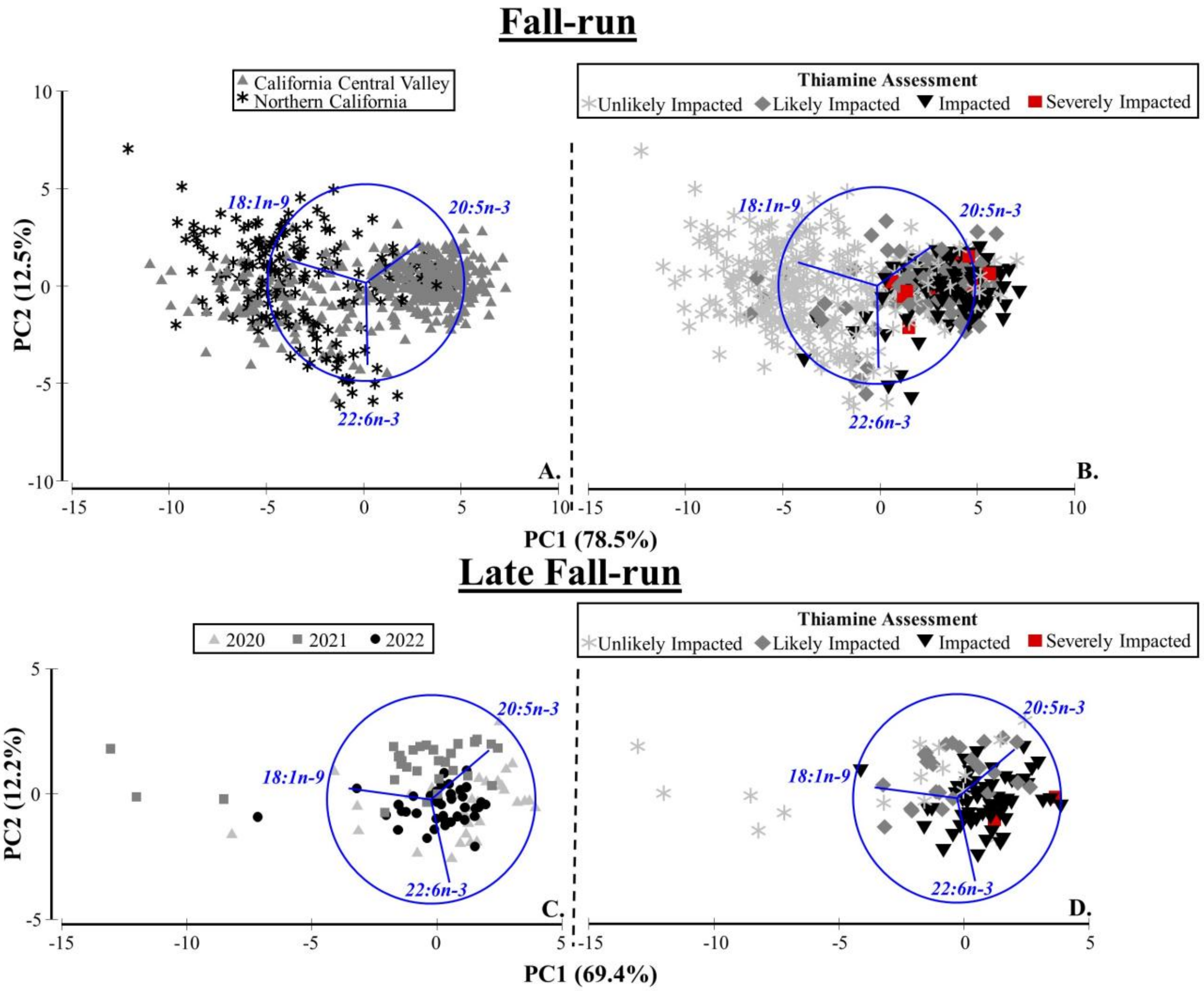

SI Figure 1. Principal component analysis (PCA) of Chinook salmon (*O. tshawytscha*) egg fatty acid signatures (FAS) collected from Northern California (NC) and California Central Valley (CCV) hatcheries in 2020-2022 based on the proportions of fatty acids (Fall-run – **A**; Late Fall-run – **C**). Vectors for 18:1n-9, 20:5n-3, and 22:6n-3 are included based on their loading values. The percent variation accounted for by each principal component (PC) is included in parentheses on the axis title. Egg FASs are plotted with their corresponding egg thiamine concentrations and assessed based on the thiamine mortality-rate thresholds (Fall-run – **B**; Late Fall-run – **D**).

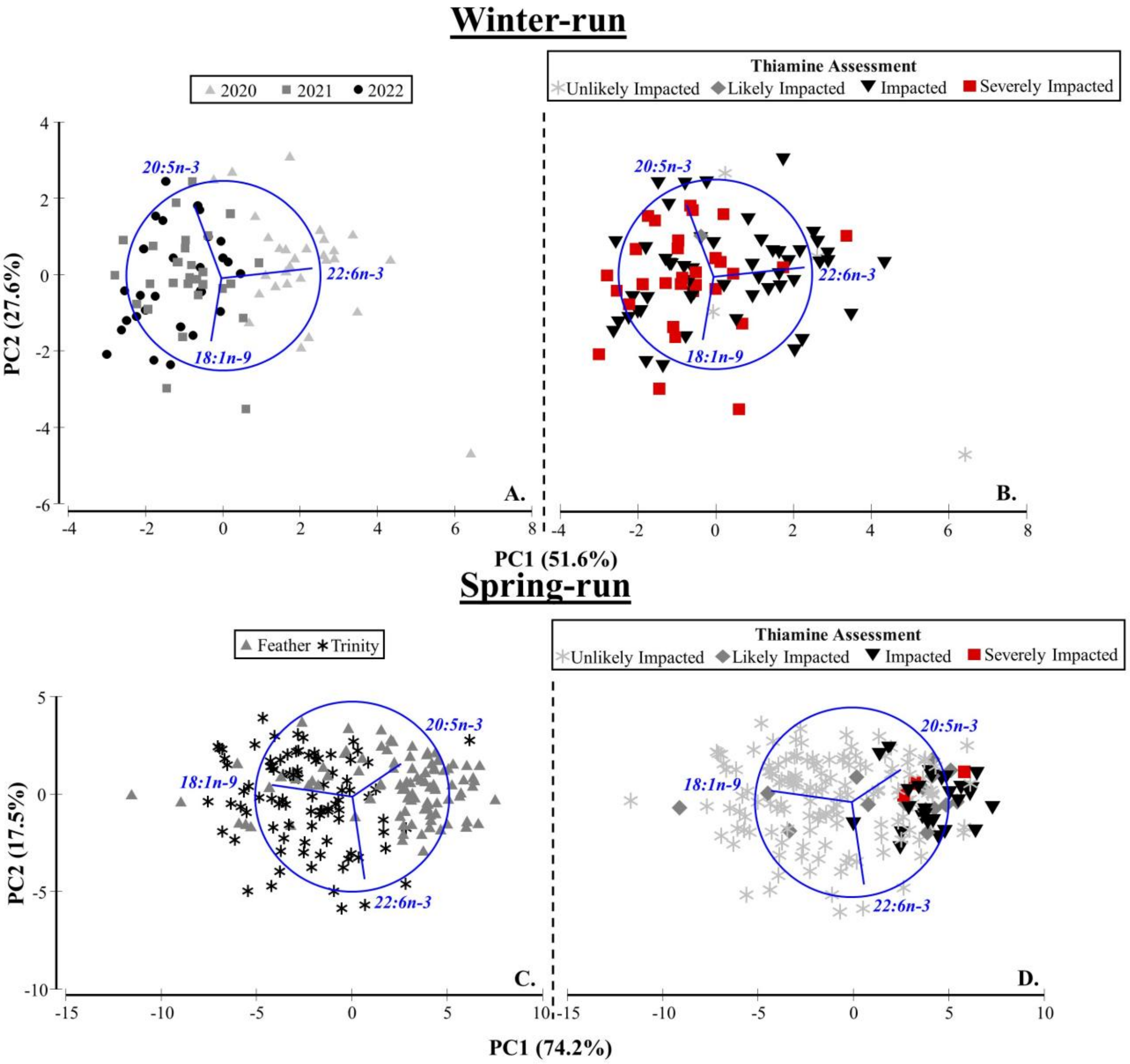

SI Figure 2. Principal component analysis (PCA) of Chinook salmon (*O. tshawytscha*) egg fatty acid signatures (FAS) collected from Northern California (NC) and California Central Valley (CCV) hatcheries in 2020-2022 based on the proportions of fatty acids (Winter-run – **A**; Spring-run – **C**). Vectors for 18:1n-9, 20:5n-3, and 22:6n-3 are included based on their loading values. The percent variation accounted for by each principal component (PC) is included in parentheses on the axis title. Egg FASs are plotted with their corresponding egg thiamine concentrations and assessed based on the thiamine mortality-rate thresholds (Winter-run – **B**; Spring-run – **D**).

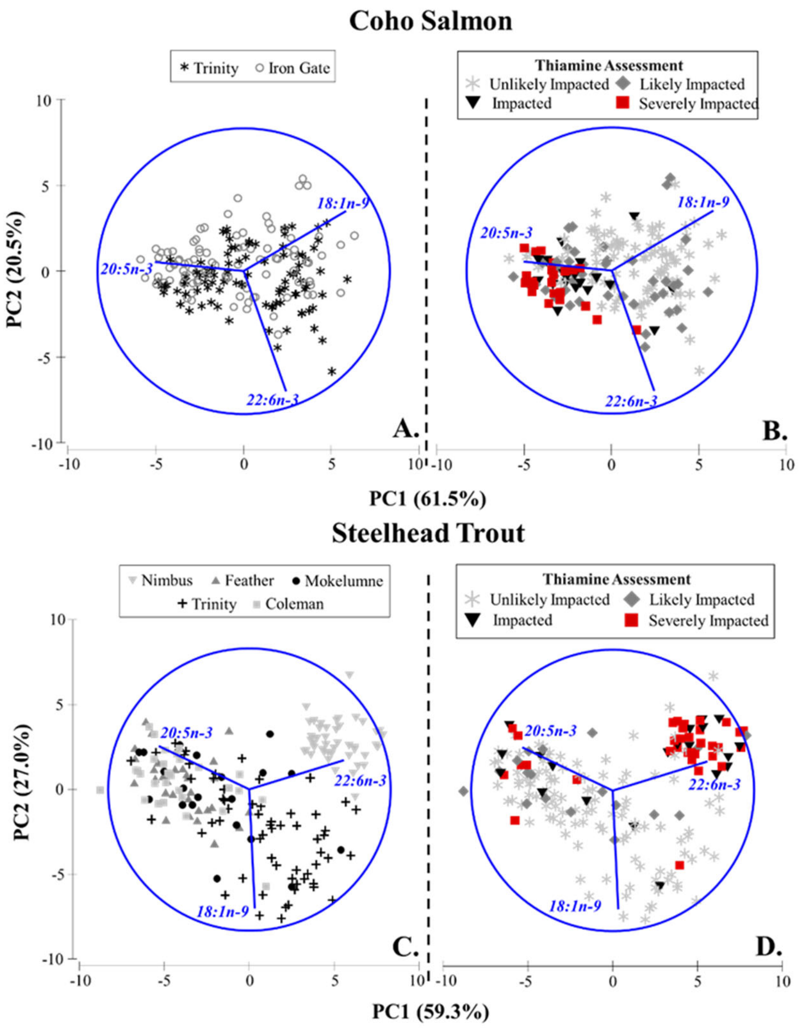

SI Figure 3. Principal component analysis (PCA) of coho salmon (*O. kisutch*; **A.**) and steelhead trout (*O. mykiss*; **C.**) egg fatty acid signatures (FAS) collected in 2020-2022 and 2021-2022, respectively. The FAS are based on the proportions of fatty acids. Vectors for 18:1n-9, 20:5n-3, and 22:6n-3 are included based on their loading values. The percent variation accounted for by each principal component (PC) is included in parentheses on the axis title. Coho (**B.**) and steelhead (**D.**) egg FASs are plotted with their corresponding egg thiamine concentrations.
